# Parity-Violating Neutron Spin Rotation in a Liquid Parahydrogen Target

**DOI:** 10.6028/jres.110.026

**Published:** 2005-06-01

**Authors:** Diane M. Markoff

**Affiliations:** North Carolina State University, Department of Physics, Raleigh, NC

**Keywords:** cold neutrons, neutron spin rotation, nucleon-nucleon interaction, parity-violation, spallation neutron source, weak hadronic interaction

## Abstract

Our understanding of hadronic parity violation is far from clear despite nearly 50 years of theoretical and experimental progress. Measurements of low-energy parity-violating observables in nuclear systems are the only accessible means to study the flavor-conserving weak hadronic interaction. To reduce the uncertainties from nuclear effects, experiments in the few and two-body system are essential. The parity-violating rotation of the transverse neutron polarization vector about the momentum axis as the neutrons traverse a target material has been measured in heavy nuclei and few nucleon systems using reactor cold neutron sources. We describe here an experiment to measure the neutron spin-rotation in a parahydrogen target (*n-p* system) using pulsed cold-neutrons from the fundamental symmetries beam line at the Spallation Neutron Source under construction at the Oak Ridge National Laboratory.

## 1. Introduction

The weak hadronic interaction in nuclear systems is not well characterized. The leading theoretical approach pictures the parity-violating nucleon-nucleon (NN) interaction as occurring through meson exchange with one strong interaction vertex and one vertex described by phenomenological weak couplings. Desplanques, Donoghue, and Holstein (DDH) estimated the nucleon-nucleon-meson weak couplings using quark model and symmetries arguments and determined a reasonable range of values including their “best guess” values for each of the seven coupling amplitudes [1]. The experimental pursuit of identifying the strength of these couplings has not produced a consistent set of data. A comprehensive discussion of the parity-violating weak hadronic interaction, including the motivation for pursuing this field of study, its role in a broader context of strong and weak quark-quark interactions, and the current status of the theoretical and experimental approaches can be found in these proceedings. See paper by W. M. Snow in this Special Issue.

An experimental and theoretical program of study was developed by the community to characterize the weak hadronic interaction in low-energy nucleon-nucleon systems. The experimental plan involves a series of measurements in two and few nucleon systems (to minimize nuclear structure effects) to determine the weak-interaction amplitudes either in the vector meson exchange model or expressed as a function of transition amplitudes between S-wave and P-wave states. The neutron spin rotation measurements in helium (five-body system) and hydrogen (two-body system) are included in this set. In these proceedings is a description of the spin rotation in liquid helium experiment, including a discussion of the preliminary measurement, the apparatus modifications being carried out and the plans for taking data. See paper by C. D. Bass in this Special Issue.

The transverse spin polarization vector is a linear combination of plus and minus helicity states, *σ*_±_. The parity-violating (PV) neutron spin rotation about the momentum axis as long-wavelength (*λ* > 1 Å) neutrons traverse the target medium arises from the spin-dependent part (*σ*_±_·**p** term) of the weak interaction forward scattering amplitude. As a result of the weak interaction, each helicity state propagates with a different phase so that the transverse polarization vector rotates. The magnitude of the rotation is directly proportional to the parity non-conserving (PNC) part of the forward scattering amplitude, *f*_PNC_(0), and is given by

$$\varphi_{\mathrm{PNC}}=4\pi \;\rho l\;f_{\mathrm{PNC}}\left(0\right)$$ (1)

where *φ*_PNC_ is the parity non-conserving neutron spin rotation, *ρ* and *l* are the density and length of the target material, respectively [2]. The forward scattering amplitude is proportional to the real part of the weak neutron-nucleus matrix element. Therefore, the size of the PV spin rotation is a direct measure of the strength of the weak nuclear interaction.

**Fig. 1:**
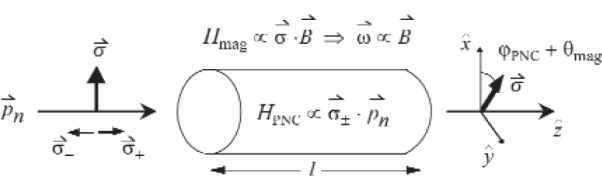
Simplified representation of the neutron spin rotation in a medium. The final integrated rotation is a combination of the parity non-conserving rotation from the target, *φ*_PNC_, and parity-conserving rotations induced by magnetic fields, *θ*_MAG_. Note that sources of magnetic field rotations are either independent of the target material or dependent upon the target material as discussed in the paper.

An axial magnetic field will rotate a transverse spin vector about the momentum axis. For example, a total spin rotation of 10 rad is expected over 1 m in the earth’s magnetic field of 50 µT. The total spin rotation from the weak interaction and the background magnetic field is presented in Fig. 1. To measure small rotations on the order of 10^−7^ rad, the background magnetic fields must be as small and homogeneous as possible. It is important to note that the flight path or time a neutron spends in a residual axial field will determine the magnitude of the total background rotation.

Using the vector meson exchange model of the weak nucleon-nucleon interaction developed by DDH and a model dependent representation of the strong interaction, Avishai and Grange calculated the neutron spin rotation through a liquid parahydrogen target and obtained [3]

$$\varphi_{\mathrm{PNC}}\left(n-p\right)=\left[-3.12\;f_{\pi }-0.23\;h_{\rho }^{0}-0.23\;h_{\omega }^{0}-2.25h_{\rho }^{2}\right]\times 10^{-6}\text{rad/m}$$ (2)

where *f*_π_ is the isovector pion exchange amplitude, *h*^0^_ρ_ and *h*^2^_ρ_ are, respectively, the isoscalar and isotensor *ρ*-meson exchange amplitudes and *h*_ω_ is the isoscalar -meson exchange amplitude. Given that *f*_π_, *h*^0^_ρ_, and *h*^2^_ρ_ are of the same order of magnitude and *h*^0^_ω_, is smaller than the others, Eq. (2) clearly demonstrates the strong dependence of *φ*_PNC_(*n-p*) on the isovector pion exchange amplitude. A measurement of the *n-p* spin rotation observable would determine *f*_π_ in a complementary way compared to another parity-odd observable in the *n-p* system, the gamma asymmetry in polarized neutron capture on protons. (For a discussion of this measurement, please see the article by S. Page et al. in this Special Issue.) The coupling *f*_π_ is of particular interest because of its long range. The dominant contribution is from the neutral current part of the weak interaction. At present, the set of low-energy weak interaction data indicates different ranges for the isovector amplitude. Using the DDH theoretical “best values,” the spin rotation observable in the (n-p) system is − 9 × 10^−7^ rad/m. Using a reduced value for *f*_π_, consistent with the parity doublet measurement in ^18^F, the prediction becomes − 6 × 10^−7^ rad/m. For a 20 cm target, a sensitivity of less than 6 × 10^−8^ radians is needed to separate these two predictions.

**Fig. 2:**
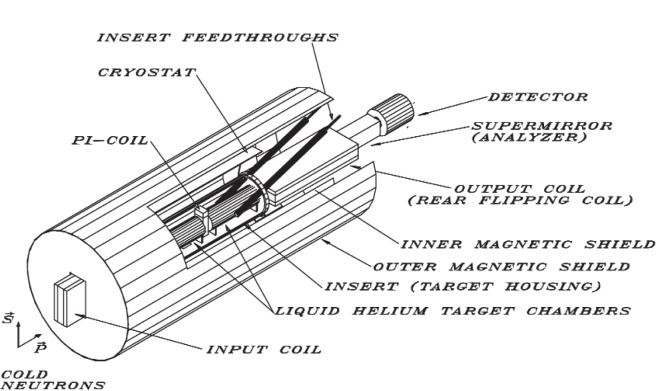
Schematic of the spin-rotation apparatus used in the liquid helium target measurement. The neutrons enter the apparatus (on the *z* axis) with an initial vertical (*x* axis) polarization. The front coil is designed to preserve the spin as the neutrons pass from a high-field region to the field-free target region inside the cryostat. Two alternately filled liquid para-hydrogen targets are separated by the central π-coil with its axis oriented along the initial vertical (*x* axis) neutron polarization. The neutrons leave the target region through the end coil that preserves the horizontal (*y* axis) component of the spin and with the final supermirror polarizer, functions as an analyzer filter. The neutron count-rate is monitored by a segmented ^3^He ionization chamber as a function of position and coarse energy bins.

The spin rotation apparatus is based on a crossed neutron polarizer and analyzer designed by Heckel et al. [4] for solid targets and is represented schematically in Fig. 2 for the liquid helium target measurement. For an ideal polarimeter, a measurement of the count rate asymmetry for analyzer alignment plus and minus is proportional to the sine of the integrated rotation angle, *ϕ*. The polarimeter is designed to minimize and separate parity-conserving (magnetic field induced) rotations from the parity-violating signal. Magnetic fields are reduced with the use of high permeability material shielding and trim coils. The front and rear coils serve to preserve the neutron spin in the transition between high field regions and the very low field (< 10 nT) target region. A more sophisticated means of performing the traditional “target in” and “target out” comparisons to remove background signals is achieved with two target positions separated by a coil that rotates the spin ideally π radians about the vertical (initial polarization) axis. As a result, rotations in front of the π-coil are negative relative to the rotations behind the π-coil. By alternately filling and emptying the target chamber in front then behind the π-coil, the parity-violating signal follows the target material and is modulated in sign between negative for target in front and positive for target in back while the background is ideally constant. A comparison of these two configurations allows for a subtraction of the constant parity-conserving background rotations to the extent that the background does not change with target position.

In a realistic apparatus, target dependent magnetic fields exist for example the diamagnetic properties of the target material alters the field in the chamber region. Target dependent neutron scattering effects introduce changes in beam divergence or path length that behave like target dependent magnetic field rotations. These two types of parity-conserving rotations will mimic the target dependent parity violating signal, cannot be subtracted away, and must be reduced below the desired sensitivity of 10^−7^ rad/m.

Compared to the liquid helium and solid targets, the scattering rate in the hydrogen target is quite high requiring more effort to control the scattering systematics. There are two states of the hydrogen molecule, H_2_, depending on the relative alignment of the proton spins: the spin 1 ortho-hydrogen and the spin 0 para-hydrogen configurations. The neutron scattering cross section is about a factor of 20 higher for the ortho configuration compared to the para configuration. In addition, at relatively high energies (*E* > 15 meV) spin-flip scattering is allowed on the ortho-hydrogen molecule which adversely affects the rotation angle. To minimize scattering systematic effects, the para-hydrogen molecule is used. Note that the neutron mean free path in para-hydrogen is about 20 cm compared with 1 m or greater in liquid helium.

We plan to mount the hydrogen spin rotation apparatus at the spallation neutron source in Oak Ridge, TN. The pulsed nature of the beam provides important advantages for characterizing systematics. With the pulsed time structure, neutrons can be counted as a function of time that corresponds to an average neutron velocity so that the faster neutrons arrive first and the slower, longer wavelength neutrons arrive later. The time structure can be used to filter the higher energy neutrons and the low-energy, long wavelength neutrons that tend to have a greater beam divergence. As can be seen from Eq. (1), the PV spin rotation is independent of neutron velocity (energy) while the scattering and magnetic field rotations are energy dependent. The background false (parity conserving) signals can be characterized by measuring the count-rate asymmetry, or spin rotation angle, as a function of energy, while a measurement of a constant PV signal as a function of time puts a convincing upper limit on these false contributions.

Significant changes to the spin rotation polarimeter from the helium apparatus include: 1) a new cryostat appropriate for a 20° liquid parahydrogen including important safety features, 2) an upgraded position sensitive detector and 3) a change in π-coil running conditions from static to dynamic.

With the danger and risks associated with known ratios of hydrogen to oxygen, extensive safety features and procedures must be instituted for a hydrogen target system and cryostat. We will put to use the experience gained with the design and certification of the 30 cm long, 20 l liquid para-hydrogen target for the np-dγ parity violation experiment. (Please see the paper by S. Page in this Special Issue.) The target will be operated at 17 K where the equilibrium ortho to para hydrogen ratio is 0.03 %. If needed, a catalyst of paramagnetic material can be used to convert the ortho-hydrogen to the para configuration. The target length is constrained by the scattering systematics and set to one mean-free-path of 20 cm.

The collection plates of the segmented ^3^He ionization chamber used in the helium measurement is divided into four quadrants providing useful neutron count-rate information in the upper, lower, right and left regions of the beam [5]. The PV signal must be independent of geometry as well as energy, and position sensitivity provides a handle on the scattering systematics. We propose to develop a neutron detector that can collect the entire beam with a 1 cm position resolution. The design combines the ^3^He conversion of neutrons to charged particles (protons and tritons) as in an ionization chamber, with the fast timing and position sensitive charge collection techniques of the MicroMegas detector. The MicroMegas technique [6] uses a wire mesh grid to locally amplify the electron charge that is subsequently detected on wire strips or pixels. Several detector modules will be placed in series along the beam axis providing 100 % collection efficiency. (A discussion of this proposal can be found by D. M. Markoff et al. in this Special Issue.)

The π-coil, smears the polarization of the neutrons in a polychromatic neutron beam, producing a count-rate asymmetry that corresponds to a rotation about one-half the true rotation angle. A neutron precesses about the π-coil magnetic field by an amount proportional to the time spent in the field. Therefore, the precession angle was inversely proportional to the neutron velocity, or linearly dependent upon the wavelength. For a continuous polychromatic beam, the coil current was optimized to rotate the majority of the neutrons by an angle as close to 180° as possible, while some neutrons are necessarily over-rotated and others are under-rotated. The measured angle was an energy averaged value of the spread in transverse components of the polarization vector. A measurement of the polarization product, *P*, gives the effective reduction in the rotation angle, that is, sin(*ϕ*) = *P* sin(*ϕ*)_ideal_. Polarization products for a monochromatic beam approach that of 90 %, compared to about 50 % on a polychromatic beam [7].

At the SNS where the neutron beam velocity is a linear function of time, we can ramp the coil current to provide a 180° rotation for each time-slice of the beam. This effectively sets the coil for a nearly monochromatic beam of width much less than 0.5 Å in neutron wavelength. Unlike in the continuous beam case, the current can be reversed during off periods of the pulsed beam incurring no additional dead time.

We have discussed the spin rotation apparatus and experimental approach to achieving a measurement sensitivity of 10^−7^ rad/m, and the systematic effects that impact this measurement. We have included a comparison to the spin rotation apparatus for a liquid helium target currently being upgraded for a second measurement and the importance of the time-structured beam at the Spallation Neutron Source for the success of the *n-p* measurement. With an expected flux of 8 × 10^9^ n/cm^2^/sec at the SNS, we can achieve a measurement with a statistical sensitivity of 2 × 10^−8^ rad in a 20 cm target with about 40 days of data, assuming scattering losses in the apparatus and spending 1/3 of the time acquiring systematic check data.
